# Human Cord Blood Derived Unrestricted Somatic Stem Cells Restore Aquaporin Channel Expression, Reduce Inflammation and Inhibit the Development of Hydrocephalus After Experimentally Induced Perinatal Intraventricular Hemorrhage

**DOI:** 10.3389/fncel.2021.633185

**Published:** 2021-04-09

**Authors:** Deepti Purohit, Dina A. Finkel, Ana Malfa, Yanling Liao, Larisa Ivanova, George M. Kleinman, Furong Hu, Shetal Shah, Carl Thompson, Etlinger Joseph, Michael S. Wolin, Mitchell S. Cairo, Edmund F. La Gamma, Govindaiah Vinukonda

**Affiliations:** ^1^The Regional Neonatal Center, Maria Fareri Children’s Hospital at Westchester Medical Center, New York Medical College, Valhalla, NY, United States; ^2^Department of Pediatrics, New York Medical College, Valhalla, NY, United States; ^3^Department of Pathology, Westchester Medical Center, New York Medical College, Valhalla, NY, United States; ^4^Department of Physiology, New York Medical College, Valhalla, NY, United States; ^5^Department of Cell Biology and Anatomy, New York Medical College, Valhalla, NY, United States; ^6^Departments of Medicine, Pathology, Microbiology and Immunology, New York Medical College, Valhalla, NY, United States; ^7^Department of Biochemistry and Molecular Biology, New York Medical College, Valhalla, NY, United States

**Keywords:** unrestricted somatic stem cells, intraventricular hemorrhage, hydrocephalus, aquaporin (AQP), choroid plexus, ependymal wall, cerebral palsy, white matter injury

## Abstract

Intraventricular hemorrhage (IVH) is a severe complication of preterm birth associated with cerebral palsy, intellectual disability, and commonly, accumulation of cerebrospinal fluid (CSF). Histologically, IVH leads to subependymal gliosis, fibrosis, and disruption of the ependymal wall. Importantly, expression of aquaporin channels 1 and 4 (AQP1 and AQP4) regulating respectively, secretion and absorption of cerebrospinal fluids is altered with IVH and are associated with development of post hemorrhagic hydrocephalus. Human cord blood derived unrestricted somatic stem cells (USSCs), which we previously demonstrated to reduce the magnitude of hydrocephalus, as having anti-inflammatory, and beneficial behavioral effects, were injected into the cerebral ventricles of rabbit pups 18 h after glycerol-induced IVH. USSC treated IVH pups showed a reduction in ventricular size when compared to control pups at 7 and 14 days (both, *P* < 0.05). Histologically, USSC treatment reduced cellular infiltration and ependymal wall disruption. In the region of the choroid plexus, immuno-reactivity for AQP1 and ependymal wall AQP4 expression were suppressed after IVH but were restored following USSC administration. Effects were confirmed by analysis of mRNA from dissected choroid plexus and ependymal tissue. Transforming growth factor beta (TGF-β) isoforms, connective tissue growth factor (CTGF) and matrix metalloprotease-9 (MMP-9) mRNA, as well as protein levels, were significantly increased following IVH and restored towards normal with USSC treatment (*P* < 0.05). The anti-inflammatory cytokine Interleukin-10 (IL-10) mRNA was reduced in IVH, but significantly recovered after USSC injection (*P* < 0.05). In conclusion, USSCs exerted anti-inflammatory effects by suppressing both TGF-β specific isoforms, CTGF and MMP-9, recovered IL-10, restored aquaporins expression towards baseline, and reduced hydrocephalus. These results support the possibility of the use of USSCs to reduce IVH consequences in prematurity.

## Introduction

Intraventricular hemorrhage (IVH) arises from rupture of immature and developing blood vessels in the germinal matrix of premature infants. IVH occurs in up to 25% of preterm neonates where 10% of events are further complicated by hydrocephalus (Campos-Ordonez et al., 2014; Koschnitzky et al., 2018). A recent NIH workshop on post-hemorrhagic hydrocephalus (PHH) reported that IVH affected neonates develop somatic growth impairment, white matter damage, motor dysfunction and neurocognitive deficiency (Koschnitzky et al., 2018). Moreover, IVH is associated with prolonged neonatal hospitalization and increased lifelong medical care costs (Christian et al., 2016). Presently, since only palliative therapies exist, mitigation of IVH related co-morbidities would reduce patient mortality and significantly reduce health care costs due to late effects of IVH on preterm newborns.

The magnitude of ventricular dilation correlates with morphological and functional damage proportional to the degree of subependymal gliosis, fibrosis and disruption of the ependymal lining (Karimy et al., 2017). Mechanistically, post hemorrhagic hydrocephalus (PHH) arises from either over secretion or impaired absorption of cerebrospinal fluid (CSF). The latter is due in part to blood obstructing arachnoid villi associated with fibro-proliferative responses, inflammation and sub-ependymal gliosis. Subtypes of ungated aquaporin water channels represent another important pathway governing CSF homeostasis, as does the glymphatic drainage system (Gunnarson et al., 2004; Zelenina, 2010; Verkman et al., 2017). AQP1 and AQP4 are associated with CSF hypersecretion and reduced absorption respectively (Verkman et al., 2017). AQP1 is expressed primarily along the choroid plexus epithelial lining and, in knockout studies using mice, the loss of AQP1 function reduced CSF production and intraventricular pressure (Oshio et al., 2005; Trillo-Contreras et al., 2019).

AQP4 channels are predominantly expressed on the surface of ependymal cells lining the lateral and third ventricles and along astrocyte foot processes surrounding capillaries (glia limitans). AQP4 channels function primarily to absorb CSF and interstitial fluid (ISF) of the brain parenchyma (Amiry-Moghaddam and Ottersen, 2003; Lehmann et al., 2004; Papadopoulos and Verkman, 2013). AQP4 knockout mice develop ventricular enlargement and increased intracranial pressure (ICP) suggesting a protective role of AQP4 in maintaining normal ventricular volume (Bloch et al., 2005; Filippidis et al., 2012). In a recent study on double-knockout mice for both AQP1 and AQP4, the preponderance of CSF accumulation was attributed to failure of AQP4 mediated CSF resorption rather than excessive AQP1 mediated CSF production (Igarashi et al., 2014; Trillo-Contreras et al., 2019). Taken together, these observations underscore the importance of aquaporin channels in CSF homeostasis.

In PHH, free hemoglobin and iron contribute to inflammation and result in increased toll-like receptor (TLR) expression which correlates with hypersecretion of CSF (Gao et al., 2014; Gram et al., 2014; Karimy et al., 2017). Further, studies with CSF show up-regulation of TGFβ isoforms (Cherian et al., 2004a) which in turn stimulate production of extracellular matrix (Whitelaw et al., 2002). However, few studies have examined the changes in TGF-β isoforms relative to the progression of subependymal gliosis and fibrosis (Kitazawa and Tada, 1994; Douglas et al., 2009; Kaestner and Dimitriou, 2013). Therefore, experiments correlating changes in TGF-β isoforms and CNS scarring after PHH would be informative.

Currently, there are no effective clinical treatments for PHH except removal of CSF by surgical diversion. Nonsurgical cell based therapies represent an emerging opportunity to prevent acute and long-term morbidity associated with PHH in the preterm neonate. Preclinical investigations of PHH created by injection of exogenous blood into the ventricles, evaluated the effects of mesenchymal stem cells (MSCs). These reports showed improved myelination, neuroprotection and less apoptosis (Liu et al., 2010; Ahn et al., 2014; Mukai et al., 2017). To expand upon these observations, our group investigated unrestricted somatic stem cells (USSCs) from human cord blood that were engineered to express the luciferase reporter gene, confirmed a stable non-teratogenic phenotype and successfully tracked USSC *in vivo* migration in a rabbit model of intraventricular hemorrhage IVH (Vinukonda et al., 2019). We selected USSCs because they release growth factors and cytokines with known neuroprotective and axonal growth promoting functions at higher levels than MSCs and for their anti-inflammatory and immunomodulatory properties. Released peptides include leukemia inhibitory factor (LIF), vascular endothelial growth factor (VEGF; Jin et al., 2000; Sun et al., 2003) and stromal cell-derived factor-1, which induces homing of neural stem cells to ischemic brain regions (Imitola et al., 2004; Kogler et al., 2005) and stimulates axonal sprouting after spinal cord injury (Opatz et al., 2009).

On the basis of USSC functions and our prior report (Vinukonda et al., 2019), we hypothesized that USSC administration would stabilizes the aquaporin water channels in the choroid plexus and ependymal wall resulting in reduced hydrocephalus after IVH. Further, we hypothesized that USSC administration would also suppress inflammation in the choroid plexus (TLR2, TLR4 and NF-kB) as well as ventricular wall matrix reorganization (TGF-β isoforms, matrix metalloproteinase-9, MMP-9), and promote anti-inflammatory cytokine (Interleukin-10; IL-10) expression which collectively would contribute to the attenuation of PhH after IVH.

## Materials and Methods

### Human Cord Blood Derived Unrestricted Somatic Stem Cell Isolation

USSCs were isolated from human umbilical cord blood mononuclear cells according to the methods of Kogler and colleagues as we have previously described (Kogler et al., 2004; Liao et al., 2014; Vinukonda et al., 2019). USSCs were transduced with a lentivirus carrying GFP-luciferase gene prepared using the lenti-viral construct, pSico PolII-eGFP-Luc2 (generously provided by Dr. Glenn Merlino at the National Cancer Institute) to enable *in vivo* identification while enabling the cells to retain their functionality (Liao et al., 2014). To ensure the rigor, reproducibility, and authentication of this biological reagent, we created multiple vials of frozen USSC stock from one batch of this isolated reagent and characterized the cells for cell-specific expression markers using microarray gene expression analysis to reconfirm fidelity with the parent USSC phenotype (Liao et al., 2014).

### Glycerol-Induced Interventricular Hemorrhage Followed by the Development of Post Hemorrhagic Hydrocephalus in Premature Rabbit Kits

Timed pregnant New Zealand white rabbits (Oryctolagus cuniculus) were purchased from Charles River Laboratories Incorporation (Wilmington, MA, USA) and premature rabbit pups were delivered by cesarean-section at E29 gestational age (term *gestation* = 32 days). Newborn pups were maintained and fed according to our previously published methods (Georgiadis et al., 2008; Chua et al., 2009). At 3–4 h of postnatal age, newborn pups were treated with 50% intraperitoneal glycerol: water (6.5 g/kg) which induced IVH in approximately 70% of all treated pups (Georgiadis et al., 2008; Chua et al., 2009; Vinukonda et al., 2019). Head ultrasound was performed at the 24 h postnatal age to determine the presence and severity of IVH [Acuson Sequoia C256, ultra-sonographic Imaging System (Siemens Corp., Washington, DC, USA)]. The grades of IVH were defined based on the ventricular volume (length, breadth and depth in coronal and sagittal views) and pups were classified as: (i) no gross IVH; (ii) moderate, gross IVH (70–150 mm^3^); or (iii) severe IVH (151–250 mm^3^) filling both ventricles completely. In our model the severe grade of IVH pups are at high risk for the development of hydrocephalus. Therefore, we included only moderate and severe IVH pups in the USSC treatment group and IVH saline group (Chua et al., 2009; Dohare et al., 2019). Hydrocephalus was defined as a ventricle area that measures more than three standard deviations (SD) above the mean for age in pups without IVH. The New York Medical College Institutional Animal Care and Use Committee (IACUC) approved all interventions.

### Anatomical Localization of Intracerebral Ventricular (ICV) Administered USSCs

USSCs were administered into the cerebral ventricle using the following coordinates from the Bregma: 1 mm posterior, 4 mm lateral and 3 mm deep (1 × 10^6^ cells in 10 μl normal saline to each ventricle) as we previously published (Vinukonda et al., 2019). We confirmed the presence and anatomical location of migrated USSCs in rabbit tissues at different postnatal ages using methods published previously (Vinukonda et al., 2019). Representative live animal bioluminescence imaging (BLI), coronal slice BLI and USSC specific immunostaining images are shown in Supplementary Figures 3G–L. Briefly, the sections were stained to identify the USSCs using anti-human nuclei (hNuc) antibody (Cat #MAB 1218, EMD Millipore, USA) and counter stained with diamidino-phenylindole (DAPI). We confirmed USSC survival and migration as previously described in our recent publication (Vinukonda et al., 2019). To determine whether USSCs could migrate from the point of administration, we tested for the presence or absence of USSCs in other organs: liver, spleen, heart and lung using USSC bioluminescence as well as specific immunostaining on representative sections made from each organ. The data confirmed no immune reactivity indicating no sustained trapping of exogenously administered USSCs in other organs (Vinukonda et al., 2019).

### Assignment of Rabbit Pups to Experimental Groups, Initial Tissue Collection and Processing for Endpoint Studies

At 24 h after birth, pups with moderate or severe IVH littermates were alternately assigned to either IVH-saline or IVH-USSC administration in birth order from each litter; the cycle was repeated until all affected pups were accounted for. Similarly, the no IVH control pups were assigned as unaffected control pups. Replicate animal experiments were conducted until the sample size for each treatment category was achieved for all assays performed. Each intervention group was targeted to receive five to six pups for analysis except for animals sacrificed solely for laser dissection of the lining of the choroid plexus. In laser dissected tissue, we used four pups per group for mRNA analysis (sp. aquaporins, TLR4, TLR2 and NF-kB) to avoid excessive wastage of animals using this technique.

We collected rabbit forebrain tissue samples from the three experimental groups of rabbit pups at postnatal days 3, 7 and 14 (Georgiadis et al., 2008; Vinukonda et al., 2010, 2019). Soon, after rabbit pups were put to death, their brains were quickly removed and cut into a single 3 mm coronal slice at the mid-septal nucleus (using a brain matrix slicer, Cat #BSRAS001-1, Zivic Instruments, Pittsburg, PA, USA) starting from the cranial end of the frontal lobe. The sub-ventricular zone (SVZ) was manually dissected under a microscope (described below) or the entire 3 mm coronal slice was directly snap-frozen on dry ice and stored in −80°C until tissues were used for RNA isolation or lysates for Western blot or ELISA analyses. The remaining part of the forebrain was directly processed, fixed and mounted as a coronal block for immunohistochemistry (IHC), cresyl violet or H&E staining described below. Supplementary Figure 1 illustrates the protocol timeline and the process of tissue harvesting.

### Hematoxylin-Eosin (H&E) Staining and Ventricle Cross Sectional Area

To measure the ventricular cross sectional area, coronal brain sections were stained by either Hematoxylin-Eosin (H&E) or cresyl violet methods as previously described (Chua et al., 2009; Gram et al., 2014; Ley et al., 2016; Vinukonda et al., 2019). After staining the images were created using an EOVS microscope with a measuring graticule using a digital scanning camera attached to the microscope (Life-Technology-Thermo Fisher Scientific, Waltham, MA, USA). To measure the cross-sectional area of the ventricles, and whole forebrain parenchyma in the three experimental groups, we used Image-J software following our previously published methods (Chua et al., 2009; Vinukonda et al., 2019). As shown in Supplementary Figure 1C, we first measured total brain area and then measured each ventricle area. To calculate brain parenchymal area, we subtracted the mean ventricular area of both ventricles from the total region. The final data are presented as mean ± SEM.

### Immunohistochemistry (IHC) and Image Analysis for AQP1 Expression

Immunohistochemical staining was performed as we previously described (Braun et al., 2007; Vinukonda et al., 2010). Briefly, the tissue was fixed in 4% paraformaldehyde in phosphate buffered saline (0.1 M PBS; pH: 7.4) for 18 h followed by cryoprotection by immersing slices in 15% sucrose in 0.1 M PBS for 24 h and then another 24 h in 30% sucrose. Finally, the cryoprotected tissues were frozen into optimum cutting temperature compound (Sakura, Japan; Cat #23-730-571, Thermo Fisher Scientific) for 20 μm sectioning and staining. The fixed sections were hydrated in 0.01 M phosphate buffered saline (PBS) and incubated with the primary antibodies overnight at 4°C followed by a secondary antibody for 1 h at room temperature. After washing, the sections were mounted with mounting media—slow fade Light Anti-fade reagent (Molecular probes, Invitrogen, CA, USA) and then visualized microscopically. AQP1 staining was performed using mouse monoclonal antibody (Cat #NB600-749, Novus Biologicals, Littleton, CO, USA) and AQP4 immunostaining was done using mouse monoclonal antibodies (Cat #ab9512, Abcam, Cambridge, UK^1^). The sections were counter stained with diamidino-phenylindole (DAPI) to identify nuclei density to facilitate structural boundaries of the brain. The fluorescence signals for immunoreactivity were imaged at 4×, 10× and 20× objectives, using a Nikon Eclipse 90i microscope a Nikon DS-Qi1Mc camera, and NISE elements AR 4.20 software (Nikon Instruments, Japan).

Multiple images of the entire choroid plexus were obtained in the lateral ventricle for AQP1 expression in all experimental groups. Each image was analyzed for AQP1 pixel intensity using Image-J software (Rasband WS, USA; NIH, Bethesda, MD, USA^2^, 1997–2016). To quantify pixel fluorescence intensity of AQP1, the image was opened in the Image-J window and a region of interest was drawn around the choroid epithelium using the software tool (Supplementary Figure 1E). The integrated pixel density was normalized to the area measured and compared across experimental groups. To calculate the mean AQP1 intensity, we averaged two to three serial sections from each rabbit pup (six pups per group). This data are presented as mean ± SEM. The investigator was blinded to the group until the analysis was completed.

### Enzyme-Linked Immunosorbent Assay for MMP-9 Expression in the Dissected Tissue Lysate

Using the coronal brain slices at the level of the mid-septal nucleus (Vinukonda et al., 2010, 2019), approximately 200 mg of frozen tissue was homogenized in 500 μl of 1× lysis buffer from 2× stock buffer provided by manufacturer (CODE #EL-lysis, Ray Biotech). The tissue homogenates were made using Minilys homogenizer (Bertin Technologies, MD, USA) and Precellys lysing kit for soft tissue homogenizing ceramic beads (1.4 mm, part #91-PCS-CK14; Bertin Technologies, MD, USA). The lysed samples were incubated for 30 min by shaking at 4°C, following spin down in a microfuge at 10,000 rpm speed for 5 min at (4°C) and the clear supernatant was transferred into a clean tube. The total protein concentration was determined using the BCA protein assay kit (Cat #23227, Pierce, Thermo Fisher Scientific, Waltham, MA, USA). Each sample was diluted into a final concentration of 250 ng/μl. Finally, 100 μl aliquots were created and stored at −80°C to avoid repeated freeze-thaw cycles until performing antibody array/ELISA testing. The assay was performed using 100 μl aliquots according to the manufacturer’s protocol for the three experimental groups at postnatal day 14. Matrix-metalloproteinase-9 (MMP-9) quantification was assessed using the manufactures protocol (Cat #QAL-CYT-1, Ray Biotech, Norcross, GA, USA); quantification was determined using the software program provided in the kit protocol. Data were presented as mean ± SD after being normalized to protein concentration.

### Quantification of Normal and Reactive Astrocyte Cell Density

To investigate the effect of USSC infusion on astrocyte cell density in coronal brain sections, we used a primary antibody (mouse monoclonal GFAP; Cat #G6171, St. Louis, MO, USA) and counter-stained sections with DAPI nuclear staining at postnatal day 14. Since the brain hemorrhage and resultant reactive gliosis is non-uniform around the ventricle (sub-ventricular and periventricular white matter) we counted all positive cells in the entire subventricular zone SVZ and periventricular zone (PVZ) area. The cell counting on fixed sections was done as described previously (Vinukonda et al., 2010). Two investigators (blinded for experimental groups) determined the cell density using Image-J software with grid. We counted four sections in each pup and five pups from each group. Data was presented in scatter plot as mean cell count (mean ± SEM).

### Sample Preparation for RNA Isolation From Laser Microdissection of Choroid Plexus

Real-time TaqMan gene expression assays for AQP1 were performed on postnatal day 3 using laser micro-dissected choroid plexus tissue sections. Briefly, the sample was embedded in optimal cutting temperature (OCT) compound, snap frozen in liquid nitrogen and stored at −80°C. Serial coronal sections were made at the level of mid-septal nucleus (18–20 μm thickness) containing the choroid plexus in the lateral ventricles cut at −20°C using a cryostat (Leica-Germany). The sections were mounted on polyethylene naftelato (PEN) membrane slides (2.0 μm Leica Microsystems 3P, Germany). Laser capture microdissection was performed using a LMD6500 Leica laser microdissector (Leica-Germany). The CP was viewed on its monitor using a 20× objective and microsamples of the CP were selected using the software tool. Laser dissected CP from both lateral cerebral ventricles are shown in Supplementary Figure 1D. The collection tube caps were preloaded with 50 μl of RLT plus buffer and immediately processed for RNA extraction.

### Microscopic Dissection of Ependymal Wall SVZ Tissue for RNA Isolation

SVZ tissue samples were harvested at the level of the mid-septal nucleus from 3 mm thick coronal slice on postnatal days 3, 7 and 14. We chose this level because the germinal matrix is noticeable at this location and is a useful landmark to reproducibly dissect homogenous tissues from all experimental groups. Slices were separated into wax-coated petri dishes containing sterile cold PBS and stabilized with sterile head pins so that the lateral ventricle could be viewed under a dissecting microscope. As shown in the Supplementary Figures 1F,G, we dissected a region of 0.5–1 mm size reflecting the ependymal wall using fine microdissection scissors along the lateral side of both lateral ventricle walls. Immediately after dissection of the tissue, samples were snap frozen in liquid nitrogen and stored it at −80°C, until further processing.

### Total RNA Isolation and cDNA Synthesis

Total RNA extraction was performed using three different isolation procedure kits to optimize extraction from various tissues quantities obtained after microdissections. Briefly, after homogenization, RNA was precipitated with 70% alcohol and purified total RNA was collected using a spin column following the manufacturer’s protocol. We have used three specific RNA isolation kits based on the types and amount of sample used for the isolation: (i) the “RNeasy-plus micro kit” (Cat #74034, Qiagen) specific for micro-dissected cryosections, was used for extraction of RNA from the laser dissected sample collected into RLT-plus buffer containing sterile Eppendorf tube. (ii) The “RNeasy micro kit” (Cat #74004, Qiagen) was used for RNA extraction tissue samples collected using microscope from the SVZ; and (iii) we used the “RNeasy mini kit” (Cat #74104) for the larger 3 mm thick total coronal slice. RNA was eluted into RNase free water, the quality and quantity were assessed using a Nano-Drop^®^ Spectrophotometer ND-2000C (Thermo Fisher Scientific, Waltham, MA, USA). The first-strand complementary DNA (cDNA) was synthesized by using 1 μg of total RNA with oligo-dT primers using the transcriptor high fidelity cDNA synthesis kit (Cat #05081955001, Roche Diagnostics, Indianapolis, IN, USA). In the case of RNA extracted from laser-dissected samples, quantity was not measured, and the total amount was used to make cDNA due to the lower quantity of total RNA from this tissue.

### TaqMan Gene Expression Assay

The TaqMan gene expression was performed using ABI quanta studio (Thermo Fisher Scientific, Waltham, MA, USA) as previously described (Vinukonda et al., 2010, 2019). The mRNA transcript levels were determined by two-step quantitative real time TaqMan PCR using the TaqMan chemistry reaction mix purchased from Roche (Cat #04913850001, Roche, Indianapolis, IN, USA). The following single tube TaqMan probe-primer mixes were used: Oc03395705_m1 (AQP4), Oc04096741_m1 (AQP1), Oc03395687_g1 (CTGF), Oc03397520_m1 (MMP-9), Oc03824728_s1 (TLR2), Oc03398503_m1 (TLR4), Oc03398424_m1 (TGFb2), Hs01086000_m1 (TGFb3), Oc03396940_m1 (IL-10), Oc03823402_g1 (GAPDH; Thermo Fisher Scientific, Waltham, MA, USA) as described previously (Vinukonda et al., 2010). Analysis was completed using the efficiency corrected ΔΔCT method and the data was presented in percentages (Livak and Schmittgen, 2001; Schmittgen, 2001).

### Statistical Methods

To determine differences in cell counts between the three groups, we used one-way ANOVA to compare treatments. Comparisons between groups on day 3, 7 and 14 were conducted using one way ANOVA using GraphPad Prism-6 (GraphPad Software, CA, USA). All *post hoc* comparisons were done using Tukey multiple comparison testing and *P*-values < 0.05 were considered significant. While gender differences are an important component of biological variation, since there is no published evidence of gender difference causing IVH and in an effort to minimize animal exploitation, we did not perform a gender-specific analysis of our data sets.

## Results

### USSCs Significantly Reduced Ventricular Enlargement After Intraventricular Hemorrhage

**Figure 1:**
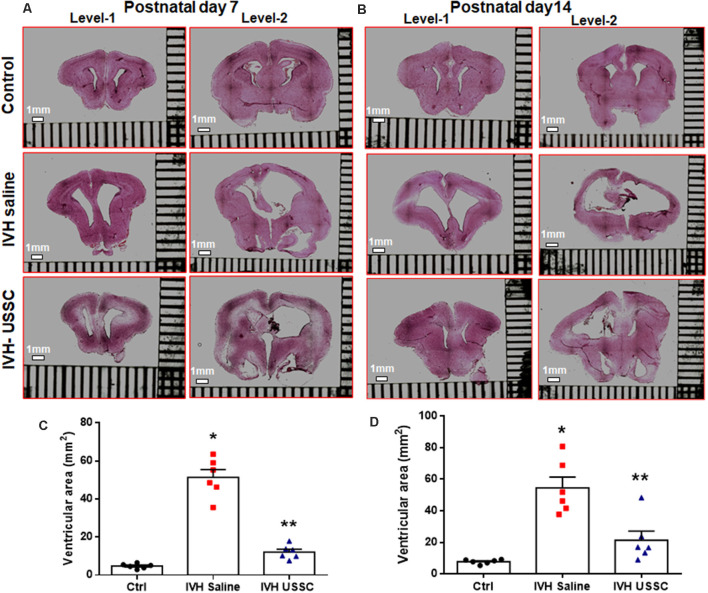
Intracerebroventricular unrestricted somatic stem cells (USSC) administration reduced cross sectional area of lateral ventricles in post-hemorrhagic hydrocephalus (PHH). **(A)** Representative Hematoxylin and Eosin (H&E) stained coronal section on postnatal day 7 at two levels (rostral forebrain: level-1 and caudal forebrain: level-2) with and without USSC administration, showing ventricular size in normal healthy control, intraventricular hemorrhage (IVH) saline control and single dose USSC (2 × 10^6^ cells/dose) injected (left panel **A**). **(B)** Ventricular area on postnatal day 14 in normal healthy control, IVH saline control and single dose USSC (2 × 10^6^ cells/dose) injected (right panel **B**). The stitched images are taken at low power for both postnatal ages from 20 μm sections. The scale bar in all images is 1 mm. Images were scanned using EVOS^®^ FL Auto Imaging System (Thermo Fisher Scientific, Waltham, MA, USA). **(C,D)** Scatter plots with bar graphs showing cross-sectional area of lateral ventricles measurement on postnatal day 7 **(C)** and 14 **(D)**. The mean cross sectional measured on two alternate sections taken from hippocampus towards the rostral side of the coronal block made at the level of mid-septal nucleus (total ventricular area is the sum of the left and right values at level-2 that are averaged from two alternate section for each pup). Each symbol in the experimental groups represent one rabbit pup (*P < 0.05 for control vs. IVH and **P < 0.05 for IVH vs. USSCs; the values represent mean ± SEM; *n* = 6 in each group for both postnatal days 7 and 14). *P*-values were derived by one-way ANOVA with Tukey’s multiple comparisons test.

We assessed the cross-sectional area of the lateral ventricles and the brain parenchymal area on coronal sections taken at the level of the mid-septal nucleus on postnatal days 7 and 14. Representative H&E images are depicted in Figure 1A for day 7 and in Figure 1B for day 14; the mean ventricular area is presented as shown in Figures 1C,D. We found a significantly higher mean ventricular area in IVH pups with PHH when compared with the no IVH controls at postnatal days 7 and 14 (mean ventricular area for day 7 was 5 ± 0.5 mm^2^ in control vs. 51 ± 10 mm^2^ in IVH; and for day 14 was 8 ± 0.6 mm^2^ in control vs. 54 ± 7 mm^2^ in IVH, *P* < 0.05 for both). Importantly, USSC administration *via* the ICV route attenuated the ventricular enlargement caused by hemorrhage at both postnatal ages 7 and 14 days (mean ventricular area was 51 ± 10 mm^2^ in IVH vs. 12 ± 4 mm^2^ in USSC injected IVH pups at postnatal day 7 and for day 14 was 54 ± 7 mm^2^ in IVH vs. 21 ± 6 IVH + USSC injected pups; Figures 1C,D, *P* < 0.05 for both). USSC-treated IVH pups showed a reduction in ventricular area (~60%) when compared to non-treated animals at 7 and 14 days. To confirm that the attenuated ventricular area was the functional effect of USSCs rather than the glycerol injected in the peritoneal cavity, we assessed ventricular cross-sectional area measurements among healthy controls (no glycerol injected, no IVH) vs. glycerol injected but no IVH rabbit pups on postnatal day 14 (Supplementary Figures 3A–D). There was no significant difference between two groups for either total brain area or for mean ventricular area on postnatal day 14 (Supplementary Figures 3C,D).

The mean total brain area (parenchyma + ventricles) was comparable across the experimental groups, whereas the brain parenchymal area alone was significantly reduced after IVH on postnatal day 14 (Supplementary Figures 2A,B, *P* < 0.05 for brain parenchyma Ctrl vs. IVH). Taken together, this data indicated that the reduction in the ventricular area after USSC injection in IVH pups is mediated by USSC stem cells and does not arise as a primary result of changes in brain parenchymal volume after PHH.

### Morphological and Histological Changes of the Lateral Ventricle Ependymal Wall and Choroid Plexus Epithelium in PHH

**Figure 2:**
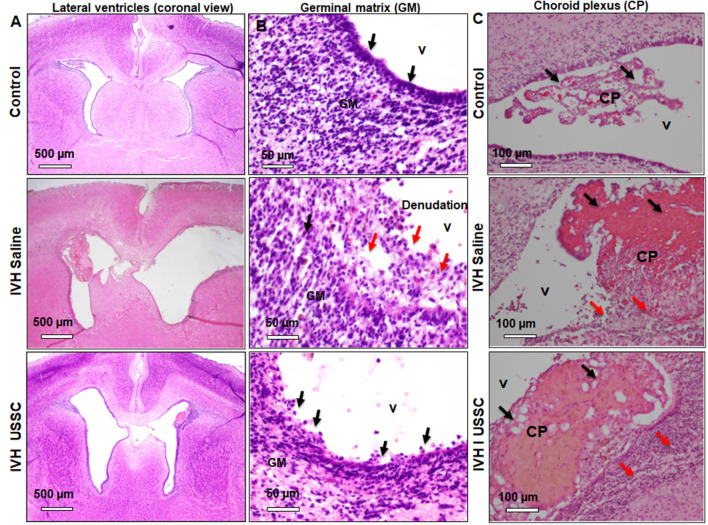
Histopathological recovery after USSC administration in germinal matrix and choroid plexus in the lateral ventricles after PHH. **(A,B)** Representative H&E stained coronal sections at the level of mid-septal nucleus after USSC administration. Note: the normal intact ependymal wall in control low power (black arrow, top panel-**A**; 4×) and high power (top panel-**B**; 20×) vs. the damaged wall after IVH (red arrow middle panel **A**; 4× and panel-**B**; 20×). Compare effects after USSC injection showing recovered germinal matrix (bottom panel **A** and **B**; 4× and 20× images). The arrow shows normal and damaged areas. The Scale bar for panel **(A)** 500 μm and for panel **(B)** is 50 μm. **(C)** Representative H&E stained coronal section with choroid plexus showing normal intact choroid epithelium in control shown in black arrow (top panel-**C**; 20×), damaged choroid plexus (CP) filled with blood shown in black arrow, histiocytic and damaged epithelium in IVH (middle image panel–**C**; 20×) compare to effects of USSC injection showing partial recovery in the CP (panel-**C**; 20× image). The arrow shows normal vs. damaged areas. The scale bar for all images is 100 μm, USSC: 2 × 10^6^ cells/dose. All images were taken using Olympus microscope model BX43F (Olympus Corporation-Life Sciences).

The morphological damage is dependent on massive RBC extravasation into the parenchyma and the lateral ventricles (physical damage) followed by subsequent secondary toxicants released by RBC degradation (cellular and molecular damage). Since the area and gross damage is variable, for consistency of comparisons, we specifically focused on the ventricle wall around the GM, CPE and CP at level-2 (multiple sections starting from hippocampus towards rostral side) with anatomically matched sections taken from the mid-septal nucleus. H&E stained coronal sections of the lateral ventricles were used to evaluate epithelial cell changes and ependymal wall integrity in the lateral ventricle walls and the choroid plexus epithelial (CPE) border. Representative images around the germinal matrix of the lateral ventricles and CPE in the lateral ventricle are depicted in Figure 2. As shown in panel **A**, low power (4×) and **B** high power (20×; top to bottom), the control pups with no IVH showed an intact multicellular dense ependymal layer and preserved CSF brain parenchymal barrier (Figures 2A,B, top panel). Whereas, in IVH pups with PHH, this ependymal layer was disrupted and detached indicating damaged CSF and brain parenchymal barriers (Figures 2A,B, middle panel). Importantly, with USSC treatment, this damage was partially recovered, and some areas showed less ependymal cell damage (Figures 2A,B, bottom panel). Further, we assessed histopathological changes on H&E stained coronal sections in CPE, as shown in Figure 2C. The choroid plexus (CP) sections from IVH pups showed prominent structural damage when compared with no IVH pups who had well-organized and preserved blood CSF barrier. The epithelial cell lining of the CP was intact in the controls (Figure 2C, top), whereas in IVH pups, the cells were reduced in number, disorganized and undergoing cell death. The cells were also flattened and lost their normal layered appearance (Figure 2C, middle). The CP was also filled with blood at multiple areas of fibrosis surrounded by histiocytes (inflammatory cells). Importantly, after USSC administration, we noted partial recovery of the integrity of the CPE border (Figure 2C, bottom).

### USSC Administration Significantly Improved Homeostasis of Aquaporin-1 Expression in the Choroid Plexus Following Experimentally Induced PHH

**Figure 3:**
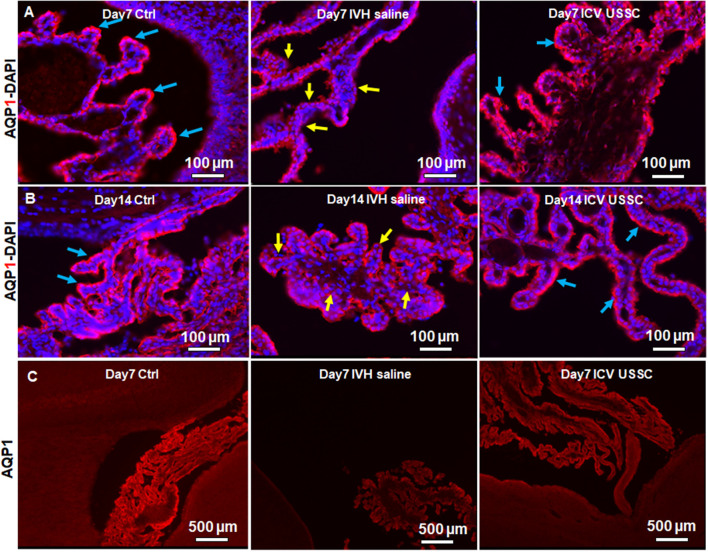
USSC administration recovered aquaporin1 (AQP1) expression in the choroid plexus after PHH. **(A)** Representative immunofluorescence image of cryosections labeled with AQP1 specific antibody (arrows showing choroid epithelium immunoreactivity) on postnatal days 7. Strong AQP1 immunoreactivity in the control (blue arrows), reduced signal (yellow arrows) in IVH followed by recovered expression (blue arrows), and images from left to right respectively at postnatal day 7. CPE (Choroid plexus epithelial cells). Images were taken at 20× objective; blue = DAPI stain, 20 μm sections. Scale bar 100 μm. **(B)** Strong AQP1 immunoreactivity in the control (blue arrows), reduced signal (yellow arrows) in IVH followed by recovered expression (blue arrows), and the images from left to right respectively at day 14. Images were taken at 20× objective; blue = DAPI stain, 20 μm sections. Scale bar 100 μm. **(C)** Representative immunofluorescence image of cryosections labeled with aquaporin1. As seen in the high power image (panel-**B** above), the strong AQP1 immunoreactivity is evident over the entire choroid plexus in the control, reduced signal in IVH followed by recovered strong AQP1 expression observed in images from left to right respectively at day 14. Images were taken at 4× objective; 20 μm sections. The scale bar is 500 μm. Fluorescence images were taken using “Nikon Eclipse 90i microscope” (Nikon Instruments, Japan).

AQP1 is a non-gated membrane transport protein primarily expressed in the choroid plexus epithelium (CPE) facing the ventricular cavities where it secrets 80% of the total CSF. We investigated whether IVH causes changes in AQP1 expression in the CPE during the progression of PHH. We performed AQP1 immunostaining on fixed coronal section on postnatal day 7 and 14 (Figure 3). AQP1 immunoreactivity decreased in early injury on day 7 when compared with no IVH controls, whereas USSC administration improved it towards normal (Figure 3A) and significantly restored levels to near normal by day 14 (Figure 3B). We also show sections with AQP1 staining (not merged with DAPI) in the CP to illustrate reduced and discontinuous AQP1 expression in IVH pups compared to control and USSC treated pups (Figure 3C); on Image-J measurements of the integrated AQP1, pixel density was reduced (*P* < 0.05, Supplementary Figure 2C). The secondary antibody Alexa-594 alone was used as negative control for AQP1 expression and showed no immunoreactivity in the CP (Supplementary Figure 2F). Pathological conditions such as IVH mediated injury can enhance short-term vulnerability of other aquaporins as well. To this end, we also examined immunostained coronal sections from IVH pups with AQP4 antibody and found weak to no immune-reactivity for AQP4 staining in this region (Supplementary Figures 2D,E).

**Figure 4:**
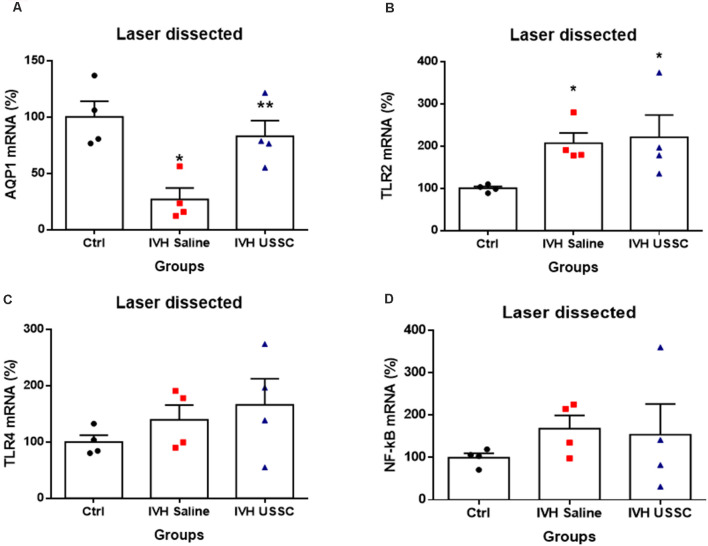
USSC administration mitigates AQP1 expression and does not suppress toll like receptors (TLRs) in choroid plexus epithelium during early IVH injury at postnatal day 3. **(A–D)** Scatter plot bar graphs showing TaqMan gene expression assay using laser dissected choroid plexus RNA at postnatal day 3. **(A)** Reduced AQP1 mRNA expression in IVH compared with healthy controls in laser dissected tissue region; after the USSC treatment, expression was recovered to normal (*P < 0.05 for Ctrl vs. IVH, **P < 0.05 for IVH vs. USSCs; *n* = 4 in each group). **(B)** Increased mRNA levels for TLR-2 after IVH independent of USSC treatment (*P < 0.05 for Ctrl vs. IVH and Ctrl vs. USSC; *n* = 4 in each group). **(C–D)** For both TLR4 and NF-kB mRNA, levels were comparable between the three interventional groups. (p –ns). The data represent mean ± SEM; for postnatal day 3.*P*-values were derived by one-way ANOVA with Tukey’s multiple comparisons test.

To test whether AQP1 mRNA expression in the CP would also change in IVH pups with and without USSC treatment, we performed real time TaqMan assays using rabbit-specific probes on cDNA made from laser dissected CP sections from coronal slices (Karimy et al., 2017). Consistent with our observation in AQP1 immunoreactivity, the AQP1 mRNA levels were also reduced (Figure 4A) in IVH pups on postnatal day 3 (100 ± 14 in control vs. 27 ± 10 in IVH) but were significantly improved by USSC administration (83 ± 14; Figure 4A, *P* < 0.05). Taken together, these results demonstrated that AQP1 expression on the choroid plexus progressively decreased in early postnatal age after IVH but was significantly restored toward normal levels after USSC administration. Importantly, the AQP4 mRNA expression in real time PCR showed high Ct (cycle threshold) detection values in CP and was not measurable after IVH.

### USSC Administration Increases Toll-Like Receptors (TLRs) and NF-KB Expression in Choroid Plexus Epithelium Following Experimentally Induced PHH

Recent studies have demonstrated that IVH derived metabolites can cause CPE inflammation resulting in CSF hypersecretion and the development of PHH. Prior studies had shown that this hypersecretion was mediated by activated TLR-NF-kB pathways (Gram et al., 2014; Karimy et al., 2017). Therefore, we tested whether IVH alters expression of TLRs and whether USSCs affected TLR levels as an index of inflammation. We performed real time TaqMan gene expression assay using rabbit specific mRNA probes for TLR2, TLR4, NF-kB using cDNA made with laser dissected CP from day 3 IVH pups with and without USSC and compared them with naïve controls (Figures 4B,D). We found significantly increased levels of TLR2 (100 ± 4 in control vs. 207 ± 24 in IVH, *P* < 0.05) and a trend toward increased TLR4 after early injury in IVH pups. With USSC administration at the early time point, both TLR mRNAs as well as the levels of the proinflammatory marker NF-kB were unaffected, Taken together the laser dissected CP mRNA expression data suggests that during the early injury phase, IVH activates TLR-NF-kB inflammation cascades. It is also evident, that a single dose of USSCs was not sufficient to suppress the TLRs’ action at an early stage of therapy and may require multiple doses of USSCs and later study time points (7–14 day) to fully characterize changes.

### USSCs’ Administration Significantly Enhanced Aquaporin-4 Expression in the Ependymal Lining of the Lateral Ventricles Following Experimentally Induced PHH

**Figure 5:**
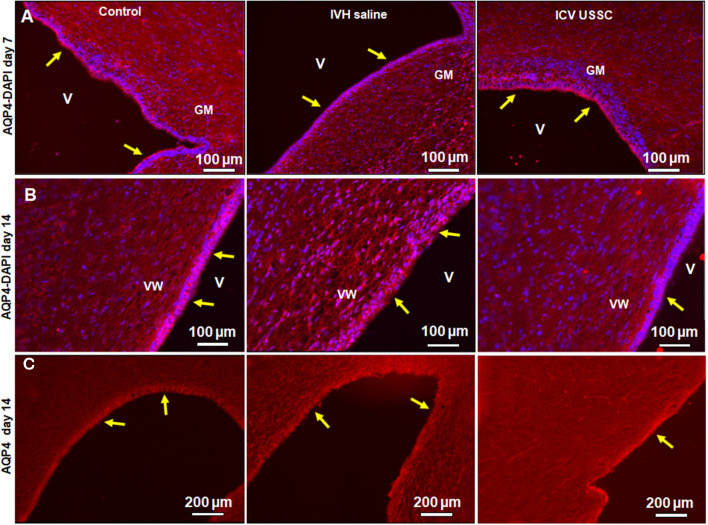
USSC administration improved aquaporin4 (AQP4) expression in the ependymal-lining layer of the ventricle after PHH. **(A)** Immunofluorescence image of cryosections labeled with AQP4 specific antibody (arrows showing immunoreactivity along the ventricular wall) at postnatal days 7. Strong AQP4 immunoreactivity was observed on postnatal day 7 in the control, reduced signal in IVH followed by recovered intensify of expression in USSC treated pups around the germinal matrix (GM); images from left to right respectively (panel **A**). Images were taken at GM region (20× objective; *n* = 5 in each group); 20 μm sections. V, ventricle; VW, ventricle wall; GM, germinal matrix. The scale bar is 100 μm. **(B)** Strong AQP4 immuno-reactivity on day 14 in the control, reduced signal after IVH followed by recovery of intensity of expression after USSC treatment and increased cellularity (blue = DAPI); images from left to right respectively. Images were taken at lateral ventricular wall (20× objective; *n* = 5 in each group); 20 μm sections. V, ventricle; VW, ventricle wall; GM, germinal matrix. The scale bar is 100 μm. **(C)** Immunofluorescence images of cryosections labeled for AQP4. The AQP4 in the ventricle wall was higher as seen in panel **(B)** in the control group and showed reduced and discontinuous signal after IVH followed by recovered to strong AQP4 expression after USSC treatment. The images were from left to right for control, IVH and IVH USSC injected pups respectively at day 14. Images were taken covering the major ventricular area at 10× objective (*n* = 5 in each group) 20 μm sections. V, ventricle; VW, ventricle wall, GM, germinal matrix. The scale bar is 200 μm. Fluorescence images were obtained using “Nikon Eclipse 90i microscope” (Nikon Instruments, Japan).

AQP4 is expressed in brain parenchyma and ependymal cells lining the lateral ventricles and facilitates the transport of excess water out of the ventricle. Increased expression of AQP4 is reported in various disease states, such as stroke and hydrocephalus (Mao et al., 2006; Paul et al., 2011). Up-regulated AQP4 is associated with excess water in the interstitial spaces, as a compensatory response to ease CSF elimination. To test whether AQP4 expression is altered in the ependymal layer lining the lateral ventricles and brain parenchyma, we first immune-stained the coronal sections using an AQP4 specific antibody on postnatal day 7 and 14. This was followed by quantification of AQP4 mRNA expression, from dissected SVZ tissue and from coronal slices at the level of the mid-septal nucleus on postnatal days 3, 7 and 14 in all the three experimental groups. The immune-reactivity for AQP4 in the lateral ventricle ependymal layer was reduced in IVH on both postnatal days 7 and 14 when compared with healthy controls (Figures 5A,B), whereas USSC administration partially improved the AQP4 immuno-reactivity (Figures 5A,B). We also observed reduced AQP4 expression on the lateral ventricular ependymal wall in IVH pups compared to control and USSC treated pups over the entire ventricular wall at low power image with single staining with AQP4 and not merged with DAPI (Figure 5C). The secondary antibody Alexa-594 alone was used as negative control for AQP4 expression. As shown in Supplementary Figures 3E,F, the ventricular wall in the lateral side and in GM region showed no immunoreactivity. It has been reported that under pathological conditions, AQP1 is also expressed in the ventricular ependymal wall as well as in the brain parenchyma. To this end, we immune-stained coronal sections for AQP1 expression in three experimental groups. We found AQP1 immuno-signals in the ependymal wall and brain parenchyma in IVH pups compared to a sparse to no expression in control and USSC treated rabbit pups on postnatal day 7 (Supplementary Figure 2G).

**Figure 6:**
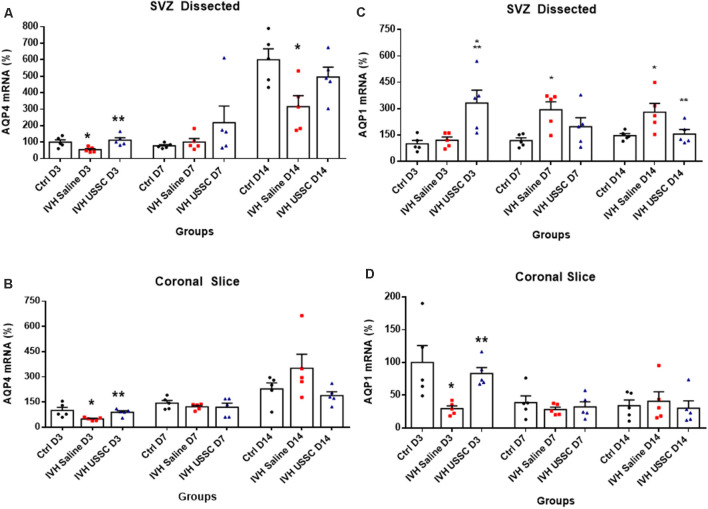
USSC administration altered the expression of the AQP4 and AQP1 mRNA in Sub-ventricular zone (SVZ) and brain parenchyma in PHH. **(A,B)** Scatter plot with bar graphs showing TaqMan gene expression assay in microscopic dissected SVZ and coronal slice tissue mRNA for AQP4 expression. **(A)** SVZ dissected tissue mRNA showed reduced expression in IVH pups compared with controls at days 3 and 14 for AQP4, whereas after USSC treatment, the AQP4 expression was elevated to normal or above IVH group at both postnatal ages (*P < 0.05 for Ctrl vs. IVH, **P < 0.05 IVH vs. USSCs, *n* = 5 in each group). The data represent mean ± SEM; for each group for three postnatal days 3, 7 and 14. *P*-values were derived by one-way ANOVA with Tukey’s multiple comparisons test. **(B)** Similar comparisons in coronal slice mRNA showed reduced AQP4 expression in IVH on day 3 that recovered to normal levels after USSCs at all time points (*P < 0.05 for Ctrl vs. IVH, **P < 0.05 IVH vs. USSCs, *n* = 5 in each group). The data represent mean ± SEM; for each group for three postnatal days 3, 7 and 14. *P*-values were derived by one-way ANOVA with Tukey’s multiple comparisons test. **(C,D)** Scatter plot with bar graphs showing TaqMan gene expression assay in dissected SVZ and coronal slice tissue mRNA for AQP1 expression. **(C)** SVZ dissected tissue mRNA on postnatal day 3 for AQP1 showed increased expression in USSC treated pups compared with controls and IVH pups (*P < 0.05 for Ctrl and **P < 0.05 IVH vs. USSCs, *n* = 6 in each group). Significantly, increased AQP1 expression occurred in IVH pups on day 7 and 14 compared to controls. After USSC, this increase was significantly reduced on day 14 (*P < 0.05 for Ctrl vs. IVH, **P < 0.05 IVH vs. USSCs, *n* = 5 in each group). The data represent mean ± SEM; for each group for three postnatal days 3, 7 and 14. *P*-values were derived by one-way ANOVA with Tukey’s multiple comparisons test. **(D)** AQP1 mRNA expression in the coronal slice RNA showed reduced AQP1 expression in IVH on day 3 and recovered levels after USSC administration (*P < 0.05 for Ctrl vs. IVH, **P < 0.05 IVH vs. USSCs, *n* = 5 in each group). Comparable AQP1 expression was observed on postnatal day 7 and 14. The data represent mean ± SEM; for each group for three postnatal days 3, 7 and 14. *P*-values were derived by one-way ANOVA with Tukey’s multiple comparisons test.

To confirm our immunostaining data, we evaluated samples for AQP4 mRNA expression in ependymal wall tissue (from dissected SVZ) and total coronal brain slices to see the regional and time dependent expression of AQP4 during critical periods of progression of hydrocephalus. In IVH pups, AQP4 mRNA was significantly reduced in both ependymal wall tissue and brain parenchyma on postnatal day 3 (100 ± 14 in control vs. 54 ± 7 in IVH for SVZ and 100 ± 18 in control vs. 49 ± 4 in IVH for parenchyma), whereas USSC administration partially improved it (113 ± 15 for USSC treated SVZ and 89 ± 7 in parenchyma; Figures 6A,B, *P* < 0.05 all pairwise comparisons). Similar trends were seen on postnatal day 7, in both dissected SVZ and brain parenchyma RNA. By postnatal day 14, AQP4 expression was significantly reduced in dissected SVZ in IVH pups but was partially improved by USSC administration (Figure 6A, *P* < 0.05).

AQP1 is mainly expressed on the choroid plexus epithelial lining and is involved in CSF secretion but under certain pathological conditions, it can also be expressed in the brain parenchyma. We next investigated AQP1 mRNA expression in the dissected SVZ and found increased levels on postnatal day 7 and 14, whereas USSC administration diminished this increase at both time points and was significant by postnatal day 14 (for day 7: 118 ± 16 control, 293 ± 45 in IVH and 197 ± 57 in USSC; for day 14: 147 ± 12 in control, 280 ± 50, 156 ± 26 in USSC treated pups; Figure 6C, *P* < 0.05). Similar results in mRNA expression were seen in coronal brain slices on day 3, the AQP1 levels decreased in IVH and improved partially by USSC infusion (for day 3: 100 ± 26 in control, 29 ± 4 in IVH and 83 ± 9 in USSC; Figure 6D, *P* < 0.05 for both). In general, for the one AQP1 expression, the CT (cycle threshold) level was 75% higher than for AQP4.

### USSC Administration Suppressed Gliosis and Fibrosis in SVZ After PHH

The subventricular zone astrocytes and the lateral ventricular wall ependymal cells arise from radial glial progenitor cells. Radial glial progenitor cell proliferation and maturation continues from early postnatal ages through maturity. An early inflammatory reaction due to hemorrhagic blood, blood components and altered TGF-β isoforms contribute to astrocytosis and scar formation, which are thought to adversely affect astrocyte lineage differentiation and proliferation during PHH. To evaluate the effects of USSC treatment, we assessed normal vs. reactive astrocyte cell counts in healthy controls and hemorrhagic pups with and without USSC administration on postnatal day 14. We found a significantly increased number of normal and reactive astrocytes (GFAP + astrocyte cell density) in IVH pups compared with controls on postnatal day 14 (for normal astrocytes in control 164.2 ± 2 vs. 305 ± 63 in IVH and reactive astrocytes in control 252 ± 41 vs. 743 ± 144 in IVH; Figure 8D, *P* < 0.05 for both). USSC administration resulted in significantly suppressed reactive astrocytosis on postnatal day 14 (mean cell density 743 ± 144 in IVH vs. 334 ± 47 in USSC treated pups; Figure 8D, *P* < 0.05).

**Figure 7:**
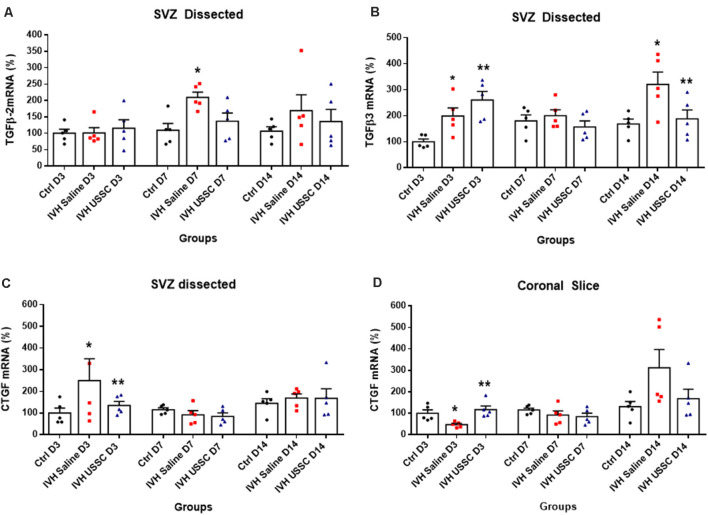
USSC administration suppressed the expression of TGF-β isoforms and connective tissue growth factor (CTGF) mRNA in SVZ in PHH. **(A,B)** Scatter plot with bar graphs showing TaqMan gene expression assay in microscopic dissected SVZ tissue for TGF-β isoforms 2 and 3 mRNA. **(A)** Increased TGF-β2 isoform mRNA was observed in IVH pups on postnatal 7th day, whereas it reduced after USSC treatment (*P < 0.05, Ctrl vs. IVH, *n* = 5 in each group). **(B)** Increased TGF-β3 expression was observed on day 3 in IVH pups with and without USSC treatment (*P < 0.05, Ctrl vs. IVH, **P < 0.05 for Ctrl vs. USSC, IVH vs USSC, Ctrl vs. USSC, *n* = 5 in each group). On postnatal day 14, TGF-β3 significantly increased in IVH pups compared to control and was reduced to normal levels after USSC treatment (*P < 0.05, IVH vs. USSC, **P < 0.05 for IVH vs. USSC, *n* = 5 in each group). The data represent mean ± SEM; for each group for three postnatal days 3, 7 and 14. *P*-values were derived by one-way ANOVA with Tukey’s multiple comparisons test. **(C,D)** Scatter plot with bar graphs showing TaqMan gene expression assay in microscopic dissected SVZ tissue and total coronal slice for CTGF mRNA levels. **(C)** CTGF mRNA was increased in IVH pups on day 3 in SVZ and suppressed to normal levels after USSC treatment and was comparable on 7th and 14th day (*P < 0.05 for Ctrl vs. IVH and **P < 0.05 for IVH vs. USSC at day 3, *n* = 5 in each group). **(D)** CTGF mRNA was increased in coronal slice on day 14 after IVH while USSC administration reduced CTGF after IVH on day 14 (*P < 0.05 for Ctrl vs. IVH and **P < 0.05 for IVH vs. USSC on day 3, *n* = 5 in each group). The data represent mean ± SEM; for each group for three postnatal days 3, 7 and 14. *P*-values were derived by one-way ANOVA with Tukey’s multiple comparisons test.

**Figure 8:**
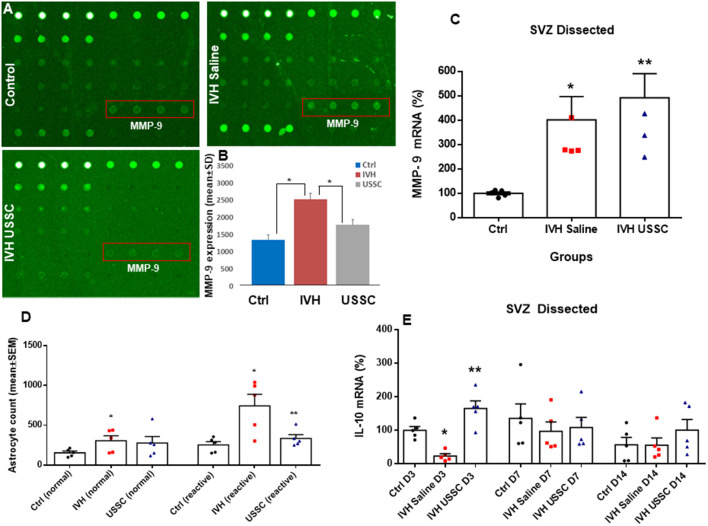
USSC administration suppressed inflammation and enhanced anti-inflammatory cytokine IL-10 mRNA expression in SVZ after PHH. **(A)** Rabbit cytokine array laser scanned images showing reduced MMP-9 expression after USSC treatment. The representative images are for MMP-9 expression in control, IVH pups with and without USSCs (boxed area = sample loaded as quadruplicates at postnatal day 14). **(B)** Bar graph shows increased MMP-9 protein expression in IVH pups compared to control pups, whereas USSC treated pups showed significantly reduced expression on postnatal day 14 (*P < 0.05 Ctrl vs. IVH as well as IVH vs. USSC pups, *n* = 5 pups in each group). The data represent mean ± SD. Assay using rabbit cytokine Array (Ray Biotech., Cat #QAL_CYT-1). **(C)** Scatter plot with bar graph for TaqMan gene expression assay in microscopic dissected SVZ tissue for MMP-9 mRNA was higher in IVH pups with and without USSC treatment on postnatal day 3 (*P < 0.05 for Ctrl vs. IVH and **P < 0.05 for IVH vs. USSC group, *n* = 5 pups in each group). The data represent mean ± SEM; for postnatal day 3. *P*-values were derived by one-way ANOVA with Tukey’s multiple comparisons test. **(D)** Total astrocyte cell count showing that normal and reactive astrocytes increased in IVH whereas after USSC treatment, a reduction in reactive astrocyte cell density was observed on day 14 (*P < 0.01 Ctrl vs. IVH and Ctrl vs. USSC for normal astrocytes and *P < 0.05 for IVH vs. Ctrl, **P < 0.05 for IVH vs. USSC both groups for reactive, *n* = 5 pups in each group). **(E)** Bar graphs show TaqMan gene expression assay in microscopic dissected SVZ tissue for IL-10 mRNA. IL-10 mRNA reduced in IVH pups compared to no IVH controls on day 3; whereas USSC injected pups with IVH showed significantly enhanced IL-10 mRNA expression (*P < 0.05 Ctrl vs. IVH, **P < 0.05 for IVH vs. USSC, *n* = 5 pups in each group). USSC treated pups showed elevated IL-10 mRNA levels on day 14 (*P < 0.05 USSC vs. other two groups, *n* = 5 pups in each group), The data represent mean ± SEM; for each group for three postnatal days 3, 7 and 14. *P*-values were derived by one-way ANOVA with Tukey’s multiple comparisons test.

Transforming growth factor-beta (TGF-β) is a multi-potent growth factor and cytokine. Altered expression of TGF-β isoforms are associated with astrocyte differentiation, scar formation and extracellular matrix tissue remodeling (Gomes et al., 2005; Hsieh et al., 2010). TGF-β dependent mechanisms are implicated in CNS fibrotic pathologies and subependymal gliosis in post hemorrhagic animal models. To investigate the changes in TGF-β expression in the SVZ, we dissected the lateral ventricle wall from the brain parenchyma and assessed mRNA levels of TGF-β isoforms (TGF-β2 and TGF-β3) including connective tissue growth factor (CTGF). CTGF, also known as CCN2, is a modular matricellular protein of the CCN family of extracellular matrix-associated heparin-binding proteins and is critically involved in wound repair and fibrotic disease (Hall-Glenn and Lyons, 2011).

After PHH, the TGF-β2 isoforms mRNA levels were increased on postnatal days 7 and 14 (for day 7: 109 ± 21 control, 209 ± 16 in IVH and 136 ± 25; Figure 7A, *P* < 0.05 for day 7) while TGF-β3 isoforms were elevated in both USSC and PHH groups on day 3 (Figure 7B, *P* < 0.05). In contrast, by postnatal day 14, TGF-β3 levels significantly increased in IVH pups but were reduced after USSC treatment (for day 3: 100 ± 11 control, 199 ± 31 in IVH and 260 ± 33 in USSC; for day 14: 168 ± 19 in control, 321 ± 47 and 187 ± 34 in USSC treated pups; Figure 7B, *P* < 0.05). Further, CTGF mRNA in the SVZ at day 3 was also significantly increased in IVH pups compared with healthy controls but was reduced in PHH after USSC treatment (for day 3: 100 ± 23 in control vs. 250 ± 90 in IVH and 135 ± 19 in USSC; Figure 7C, *P* < 0.05). While in the brain parenchymal, RNA was reduced for CTGF expression on postnatal day 3 (100 ± 16 control, 47 ± 6 in IVH and 117 ± 17 in USSC (Figure 7D, *P* < 0.05). Immune-reactivity for CTGF around the lateral ventricle showed similar patterns and was reduced after USSC treatment (data not shown). This data suggests early fibrosis after post injury in the ependymal wall rather than brain parenchymal area.

Further, we also evaluated whether USSC administration had any effect on the expression of extracellular matrix (ECM) inflammatory scar protein MMP-9. MMPs are primary components of neuro-inflammation, tissue reorganization and blood-brain barrier (BBB) disruption proteins (Rosell et al., 2006; Vella et al., 2015). Therefore, we assessed the expression of MMP-9 in the coronal lysate from healthy controls and hemorrhagic pups with and without USSC treatment at postnatal day 14, using a cytokine array assay. We found a significant increase in MMP-9 protein expression in IVH pups compared to normal healthy controls, whereas USSC administration significantly reduced MMP-9 protein expression (Figures 8A,B, *P* < 0.05). Of note, the elevation in MMP-9 mRNA expression in SVZ on postnatal day 3 was comparable in IVH pups whether they received USSC treatment or not (100 ± 6 in control, 401 ± 95 in IVH and 491 ± 98 IVH USSC; Figure 8C, *P* < 0.05 vs. control). This early response can be attributed to ependymal and parenchymal damage as well as an immediate response of exogenous USSCs to the new pathological environment. In contrast, on postnatal day 14, the increase of MMP-9 was significantly reduced after USSC treatment compared with IVH saline controls (Figure 8B). Taken together, the MMP-9 data suggests that active ECM remodeling exists early in hemorrhage and that USSCs produce an anti-inflammatory effect by day 14 which may contribute to less structural remodeling.

To further evaluate anti-inflammatory activity of USSCs, we investigated steady-state levels of the anti-inflammatory cytokine IL-10 mRNA in the three experimental groups on postnatal days 3, 7 and 14. We observed significantly reduced IL-10 mRNA levels in hemorrhagic pups compared to normal healthy controls on postnatal day 3. After ICV USSC administration, there was a significant increase on postnatal day 3 above control and IVH groups (100 ± 11 in control, 24.59 ± 7 in IVH and 165 ± 41 IVH USSC; Figure 8E, *P* < 0.05).

## Discussion

This is the first study evaluating the effects of USSCs on the density of aquaporin water channels 1 and 4 in the choroid plexus epithelium and lateral ventricular ependymal wall after IVH-induced hydrocephalus. We demonstrated that after experimentally induced IVH, USSC administration attenuated ventricular cross sectional area dimensions on postnatal ages 7 and 14 days, reduced cell infiltration and reduced denudation of ependymal cells from the lateral ventricle walls and choroid plexus (Figures 2B,C). Moreover, USSCs restored the reduced AQP1 immunoreactivity of the choroid plexus back up towards baseline (Figure 3). By comparison, in the SVZ, the converse pattern occurred where USSCs lowered the elevated AQP1 mRNA on days 7 and 14 after PHH back down toward normal (Figure 6C). After PHH, reduced levels of AQP4 immunoreactivity increased along the ependymal wall lining of the lateral ventricles and in the SVZ to nearly normal by day 14 after USSC treatment (Figures 5A,B and Figure 6A). Viewed together, our AQP1 and AQP4 data correlates with a reduced magnitude of hydrocephalus after USSC treatment consistent with improved CSF fluid homeostasis. This is significant as aquaporin’s are ungated transporters that function passively moving water in proportion to their cell surface density. Our observations do not preclude other mechanisms of CSF resorption *via* the glymphatic system or *via* arachnoid villi. Evaluation of those processes will be conducted in future work.

Inflammation is a key determinant of CNS injury where PHH is associated with generalized neuroinflammation (Del Bigio et al., 2003; Deren et al., 2009). PHH is characterized as a nonspecific reactive proliferation of astrocytes and microglia arising in part from lysis of RBCs followed by release of heme and iron (Strahle et al., 2014). PHH-induced inflammation is associated with subependymal gliosis, fibrosing arachnoiditis and meningeal fibrosis (Cherian et al., 2004a, b). We found that mRNA expression of TGF-β2 and TGF-β3 increased in the SVZ after PHH and that USSC infusion attenuated the expression of these inflammatory isoforms compared to untreated PHH controls (day 14; Figures 7A,B). USSCs also reduced the ECM inflammatory scar protein MMP-9 but not its mRNA (Figures 8B,C) and the density of reactive astrocytes (Figure 8D). The elevation of the anti-inflammatory cytokine IL-10 in both the SVZ and CP following USSC administration (Figure 8E) was consistent with this moderating pattern.

CTGF can cooperate with TGF-β to induce sustained fibrosis (Mori et al., 1999) and to exacerbate extracellular matrix production in association with other fibrosis-inducing conditions (Brigstock, 2010). The anti-inflammatory effects of USSCs were also evident as a reduction in CTGF mRNA in the SVZ on day 3 (Figure 7C) and in coronal slices of the brain parenchyma on day 14 (Figure 7D) as compared to elevated levels after IVH, respectively. TLR and NF-kB pathway mediated inflammation were not significantly altered by USSCs on day 3 (Figures 4C,D) suggesting an absent or time-dependent effect or perhaps, an insufficient quantify of cells. Taken together, our observations support an interpretation that USSCs contribute to an anti-inflammatory and regenerative process after injury.

USSCs are novel stem cell that possess significant highly anti-inflammatory and immunomodulatory capacities that have not been investigated in any IVH models of PHH. The earliest preclinical animal studies with PHH focused on mesenchymal stem cells (MSCs) in neonatal and adult models where, unlike our endogenous blood model, the injury was established by injection of exogenous blood (Ahn et al., 2013, 2014; Zhu et al., 2014). Human cord blood derived USSCs are more primitive than MSCs and do not express HLA phenotypic markers that will cause rejection in xenographic transplantation. Putative additional advantages over MSCs are that USSCs possess a higher regenerative and neuroprotective potential as shown in multiple CNS injury models (Kogler et al., 2004; Vinukonda et al., 2019). Beyond these findings, we selected USSCs because they release multiple growth factors and cytokines (Kogler et al., 2005; Mukai et al., 2017). These factors are likely to be involved in cell lineage and differentiation of multiple CNS progenitor cells in the subventricular zone both during development and in the CNS repair after injury (Hatzistergos et al., 2010; Ko et al., 2018). The current report extended our prior observations that demonstrated migration of USSCs to areas of injury and less severe hydrocephalus plus improved motor function (Vinukonda et al., 2019).

Our choice of a rabbit model of PHH was based on a range of pragmatic considerations. Rabbits have a gyrencephalic brain with a perinatal growth plan similar to newborn infants. Moreover, both are vulnerable to vascular injury immediately after birth leading to IVH and development of PHH (Ballabh, 2010, 2014). The pups are large and easy to work with and most conveniently, show glycerol-induced spontaneous rupture of the germinal matrix vasculature creating a reproducible form of IVH (Georgiadis et al., 2008; Chua et al., 2009). Significantly, PHH injury in the CNS of rabbit pups mimics cellular and behavioral effects analogous to observations in human infants including reduced myelination, ventricle dilation and subsequent behavioral deficits and motor impairments (Chua et al., 2009; Vinukonda et al., 2010).

The limitations of this study include the fact that we did not directly measure the rate of CSF turnover and so, could not parse the contributions of the other routes of CSF elimination/production in homeostasis. An additional limitation is that we identified beneficial correlations, yet, we do not know precisely what factors are induced or released by USSCs to produce the observed effects or what mechanism of release exist or how they are activated. Lastly, due to the complexities of the mechanisms examined, we elected only one dose of cells and one timing of the intervention to enable a discovery focused on putative pathways; optimized dosing will be examined in future works as the current report is clearly encouraging as a potential therapy. Other long-term transplant safety concerns, such as tumor formation, chronic rejection, etc. have not been reported thus far but will also be addressed in the future. Finally, AQ5 plays a role primarily in the generation of saliva, tears and pulmonary secretions (Sveinsdottir et al., 2014). To maintain focus in this report, it was neither studied by us in the context of CSF production nor were other channels: AQ0, AQ2, or AQ6 but are now considerations for future work on this topic.

The development of PHH is a common problem in premature infants and remains a major public health concern in the United States and around the world due to its catastrophic impact on long-term neurodevelopmental outcomes. Cell-based therapy is a promising therapeutic modality. USSCs may someday prove to be an alternative as a therapeutic approach in preterm neonates with IVH.

In conclusion, this is the first mechanistic report using human cord blood derived USSCs in the treatment of experimentally induced IVH and PHH. USSCs demonstrated anti-inflammatory effects, restored aquaporin channel expression, and mitigated the severity of PHH. Overall, these results support the continued investigation of the reparative qualities of human cord blood derived USSCs as therapy for severe IVH and for attenuation of PHH that may someday lead to clinical use.

## Data Availability Statement

The raw data supporting the conclusions of this article will be made available by the authors, without undue reservation.

## Ethics Statement

The animal study was reviewed and approved by the New York Medical College Institutional Animal Care and Use Committee (IACUC) approved all interventions.

## Author Contributions

GV: conception, design, performed experiments, assembly, analysis, interpretation of the data, and wrote the manuscript. EL and MC: conception, data interpretation, manuscript writing, financial support, and approval of the manuscript. SS, GK, CT, EJ, and MW: experimental and statistical methods, review the manuscript. YL and LI: USSC isolation, labeling and BLI imaging. FH, DP, AM, and DF: animal care, sample collection, immunostaining, cell count, neurobehavioral study, cell count, and imaging. All authors contributed to the article and approved the submitted version.

## Conflict of Interest

The authors declare that the research was conducted in the absence of any commercial or financial relationships that could be construed as a potential conflict of interest.

## Footnotes

^1^www.abcam.com

^2^http://imagej.nih.gov/ij/

## Supplementary Material

SUPPLEMENTARY FIGURE 1 Diagrammatic representation of methods and samples collection procedures. **(A)** The image showing premature rabbit pups delivered from New Zealand, white rabbit (at E29 gestational age, term 32 days). Image showing eight healthy and similar size premature rabbit pups. **(B)** Diagrammatic representation of experimental protocols performed at different postnatal ages. At 3–4 h of postnatal age, newborn pups were treated with 50% intraperitoneal glycerol: water (6.5 g/kg) to induce cerebral hemorrhage. At 24 h of age single dose USSC was injected (1 × 10^6^ cells/dose in each lateral ventricle. The samples for immunohistochemistry (IHC), PCR and lysate preparation were collected on postnatal days 3, 7 and 14. The ventricular cross sectional area was measured on postnatal day 7 and 14. **(C)** Representative of cresyl violet stained coronal section used for measuring total brain area (dotted lines) and ventricle area (marked with continuous line). The coronal sections were scanned using EVOS imaging microscope system (XL core 1000 auto scanner, Thermo Fisher Scientific, Waltham, MA, USA). The mean cross sectional was measured at the level of mid-septal nucleus. The mean cross sectional area was measured on two alternate sections taken from the hippocampus towards the rostral side from the coronal block made at the level of the mid-septal nucleus (total ventricular area is sum of the left and right at level-2 and then averaged for two alternate sections for each pup); 20 μm coronal sections. LV, left ventricle; RV, right ventricle. **(D)** H&E stained representative coronal section showing both lateral ventricles with choroid plexus. The choroid plexus was laser dissected for RNA isolation. **(E)** Immunofluroscence stained coronal section on slide showing choroid plexus immune-signal (selected with yellow dotted lines) stained for AQP1 expression and measured for pixel intensity using image-J software. The mean pixel intensity was normalized with area measured for each image. **(F–H)** Representative images for thick coronal slice and section on slide stained with H&E and AQP4 antibody. The direct measurements of thick coronal slice **(F)** and H&E stained section labeled ependymal wall dissected (dotted lines in red **(G)** represents 0.5–1 mm thick microscopic, dissected tissue) and dense AQP4 expression on the ependymal wall **(H)**.

SUPPLEMENTARY FIGURE 2 **(A,B)** Scatter plot with bar graph showing total brain area and parenchymal area at postnatal day 14. **(A)** Comparable total brain area in experimental groups on postnatal day 14 in normal healthy control, IVH saline control and IVH USSC injected pups (single dose USSC (2 × 10^6^ cells/dose). Scatter plot with bar graphs shows total brain area measured on two alternate sections taken from the hippocampus towards the rostral side from the coronal block made at the level of mid-septal nucleus. **(B)** Reduced brain parenchymal area in IVH pups on postnatal day 14. Scatter plot with bar graphs shows total brain parenchymal area that was derived by subtraction of lateral ventricular cross sectional area from total brain area (ventricular cross sectional area data from Figure 1B and total brain area data from supplement Figure 2A). Both the total brain area and the ventricular cross sectional area are measured on two alternate sections taken from the hippocampus towards the rostral side from the coronal block made at the level of mid-septal nucleus). Each symbol in the experimental groups represent a rabbit pup (**P* < 0.05 for control vs. IVH; the values represent mean ± SEM; *n* = 6 in each group for both postnatal day 14). *P*-values were derived from one-way ANOVA with Tukey’s multiple comparisons test. **(C)** Scatter plot showing mean expression for AQP1 pixel intensity measured using Image-J software (**P* < 0.05 for control vs. IVH and IVH vs. USSCs; the data represent mean ± SEM, *n* = 6 controls, 4 IVH saline and 4 IVH USSC). Each symbol in the experimental groups represent a single rabbit pup. *P*-values were derived by one-way ANOVA with Tukey’s multiple comparisons test. **(D,E)** Representative immunofluorescence images of cryosections labeled with AQP4. Weak to no AQP4 expression in the choroid plexus (CP); 20 μm sections. The scale bar is 100 μm. **(F)** Representative immunofluorescence image of cryosections labeled with negative control Donkey anti-mouse secondary antibody in CP. No AQP1 signal is seen with secondary antibody alone. Secondary antibody, Alexa-Flour 594, donkey anti-mouse (Cat #A21203, Invitrogen Thermo Fisher Scientific, Waltham, MA, USA). Highly cross-adsorbed secondary antibody with minimal cross reactivity to rabbit tissue20 μm sections. The scale bar is 100 μm. **(G)** Representative immunofluorescence image of cryosections labeled with AQP1 in lateral ventricular ependymal wall. Weak to no AQP1 expression in the lateral ventricular wall around the germinal matrix (GM). All images were taken at 20× objective; blue = DAPI stain; 20 μm sections. The scale bar is 100 μm.

SUPPLEMENTARY FIGURE 3 **(A,B)** Representative hematoxylin and eosin (H&E) stained coronal section on postnatal day 14 at forebrain level-2 with and without glycerol injected no IVH pups. The total brain area and ventricular area was comparable in normal healthy control vs. glycerol injected no IVH control. The stitched images taken at low power for both postnatal ages; 20 μm sections. The scale bar is 1 mm. **(C,D)**. Representative scatter plot with bar graphs shows cross-sectional area of lateral ventricles measurement **(C)** and total brain area **(D)** on postnatal day 14. The mean cross sectional measured on two alternate sections taken from the hippocampus towards the rostral side from the coronal block made at the level of the mid-septal nucleus (total ventricular area is sum of the left and right at level-2 and averaged for two alternate section for each pup). Each symbol in the experimental groups represent a rabbit pup (no significant differences in healthy control vs. glycerol no IIVH control; the values represent mean ± SEM; *n* = 4 in each group). **(E,F)** Representative immunofluorescence image of cryosections labeled with negative control for AQP4 Donkey anti-mouse secondary antibody in lateral ventricle wall. No AQP4 signal with secondary antibody alone cryosections stained on the lateral ventricle on the lateral side **(E)** and ventricle wall around the GM **(F)**. Secondary antibody, Alexa-Flour 594, donkey anti-mouse (Cat #A21203, Invitrogen Thermo Fisher Scientific, Waltham, MA, USA). Highly cross-adsorbed secondary antibody with minimal cross reactivity to rabbit tissue. 20 μm sections. The scale bar is 100 μm. **(G–J)** Bioluminescence live image (BLI) of USSC administration by intracerebroventricular injection in IVH premature rabbit pups. **(G)** Representative BLI live image for IVH pup after single dose of USSC (right image) and no USSC injected negative control pup (left image). The strong red fluorescence signal indicates highest USSC density at postnatal day 3 (2 × 10^6^ cells/dose). **(H)** BLI image showing dispersed USSC over postnatal day 7 and day 14 **(H)**. **(I,J)** Bioluminescence (BLI) and anatomical structures. **(I)** Image of brain coronal slice from day 3 IVH USSC injected pups, showing strong BLI signal **(I)**, and the IVH- saline control pup no USSC injected coronal slice show no BLI signals. **(J)** Coronal thick slices of brain showing blood in the ventricles. **(K,L)** Representative Immunofluroscence images of cryosections for tracking intracerebral ventricular (ICV) delivered USSC stem cells. **(K)** Using a human-specific nuclear antibody the illustration shows the USSCs (green) and rabbit cells (DAPI—blue in **L**). The data indicates healthy live cells in the rabbit brain parenchyma. Note: DAPI stains both rabbit and Human cells whereas human-nuclear antibody stains only human USSC cells. All images were taken at 20× objective; blue = DAPI stain; 20 μm sections. The scale bar is 100 μm.

## Abbreviations

IVH: Intraventricular hemorrhage
PHH: Post-hemorrhagic hydrocephalus
USSC: Unrestricted somatic stem cells
SVZ: Sub-ventricular zone
AQP1: Aquaporin 1
AQP4: Aquaporin 4
TGF-β: Transforming growth factor beta
IL-10: Interleukin-10
MMP-9: Matrix-metalloproteinase-9.
