# Supplementary figures and images for: Human Cord Blood Derived Unrestricted Somatic Stem Cells Restore Aquaporin Channel Expression, Reduce Inflammation and Inhibit the Development of Hydrocephalus After Experimentally Induced Perinatal Intraventricular Hemorrhage
Source: Front Cell Neurosci. 2021 Apr 9;15:633185. doi: 10.3389/fncel.2021.633185 (PMC8062878; doi:10.3389/fncel.2021.633185)

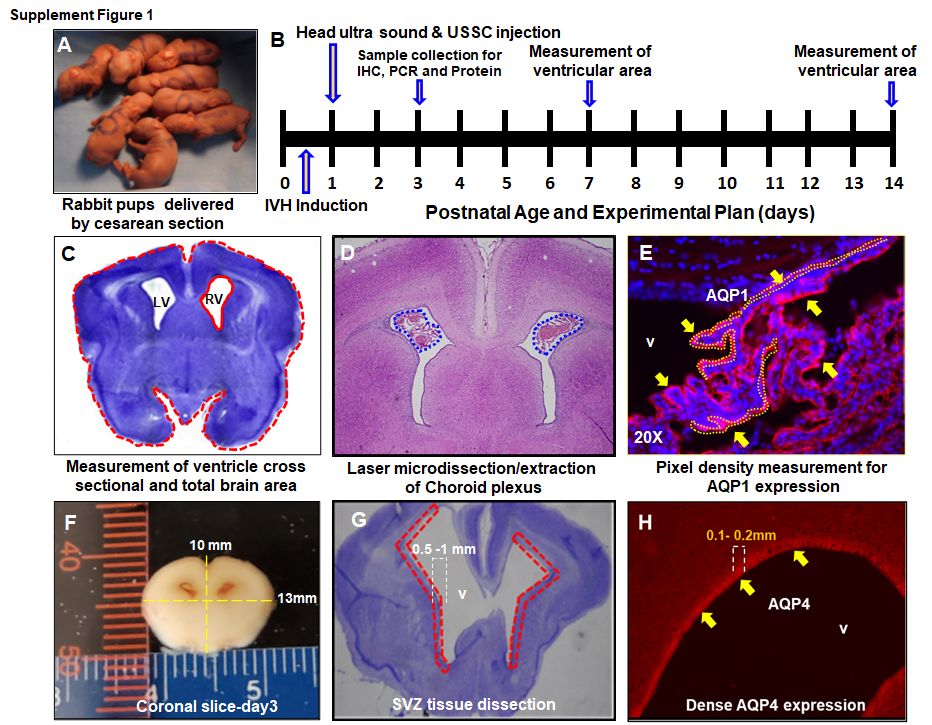

Supplement: SUPPLEMENTARY FIGURE 1 — Diagrammatic representation of methods and samples collection procedures. (A) The image showing premature rabbit pups delivered from New Zealand, white rabbit (at E29 gestational age, term 32 days). Image showing eight healthy and similar size premature rabbit pups. (B) Diagrammatic representation of experimental protocols performed at different postnatal ages. At 3–4 h of postnatal age, newborn pups were treated with 50% intraperitoneal glycerol: water (6.5 g/kg) to induce cerebral hemorrhage. At 24 h of age single dose USSC was injected (1 × 10^6^ cells/dose in each lateral ventricle. The samples for immunohistochemistry (IHC), PCR and lysate preparation were collected on postnatal days 3, 7 and 14. The ventricular cross sectional area was measured on postnatal day 7 and 14. (C) Representative of cresyl violet stained coronal section used for measuring total brain area (dotted lines) and ventricle area (marked with continuous line). The coronal sections were scanned using EVOS imaging microscope system (XL core 1000 auto scanner, Thermo Fisher Scientific, Waltham, MA, USA). The mean cross sectional was measured at the level of mid-septal nucleus. The mean cross sectional area was measured on two alternate sections taken from the hippocampus towards the rostral side from the coronal block made at the level of the mid-septal nucleus (total ventricular area is sum of the left and right at level-2 and then averaged for two alternate sections for each pup); 20 μm coronal sections. LV, left ventricle; RV, right ventricle. (D) H&E stained representative coronal section showing both lateral ventricles with choroid plexus. The choroid plexus was laser dissected for RNA isolation. (E) Immunofluroscence stained coronal section on slide showing choroid plexus immune-signal (selected with yellow dotted lines) stained for AQP1 expression and measured for pixel intensity using image-J software. The mean pixel intensity was normalized with area measured for each image. (F–H) Repres [file Image_1.tif]

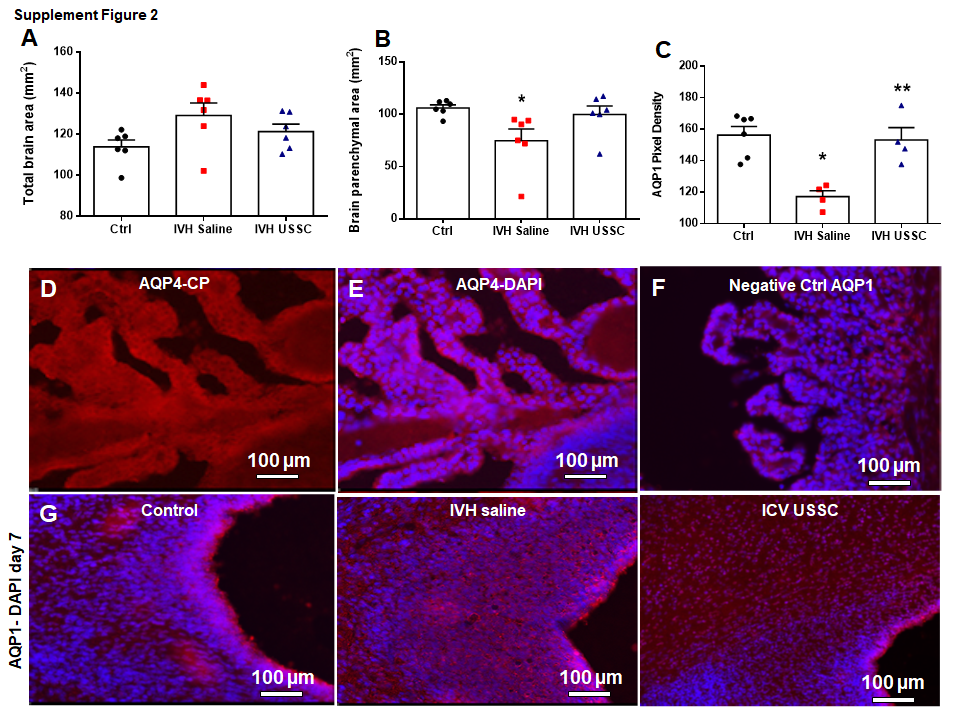

Supplement: SUPPLEMENTARY FIGURE 2 — (A,B) Scatter plot with bar graph showing total brain area and parenchymal area at postnatal day 14. (A) Comparable total brain area in experimental groups on postnatal day 14 in normal healthy control, IVH saline control and IVH USSC injected pups (single dose USSC (2 × 10^6^ cells/dose). Scatter plot with bar graphs shows total brain area measured on two alternate sections taken from the hippocampus towards the rostral side from the coronal block made at the level of mid-septal nucleus. (B) Reduced brain parenchymal area in IVH pups on postnatal day 14. Scatter plot with bar graphs shows total brain parenchymal area that was derived by subtraction of lateral ventricular cross sectional area from total brain area (ventricular cross sectional area data from Figure 1B and total brain area data from supplement Figure 2A). Both the total brain area and the ventricular cross sectional area are measured on two alternate sections taken from the hippocampus towards the rostral side from the coronal block made at the level of mid-septal nucleus). Each symbol in the experimental groups represent a rabbit pup (*P < 0.05 for control vs. IVH; the values represent mean ± SEM; n = 6 in each group for both postnatal day 14). P-values were derived from one-way ANOVA with Tukey’s multiple comparisons test. (C) Scatter plot showing mean expression for AQP1 pixel intensity measured using Image-J software (*P < 0.05 for control vs. IVH and IVH vs. USSCs; the data represent mean ± SEM, n = 6 controls, 4 IVH saline and 4 IVH USSC). Each symbol in the experimental groups represent a single rabbit pup. P-values were derived by one-way ANOVA with Tukey’s multiple comparisons test. (D,E) Representative immunofluorescence images of cryosections labeled with AQP4. Weak to no AQP4 expression in the choroid plexus (CP); 20 μm sections. The scale bar is 100 μm. (F) Representative immunofluorescence image of cryosections labeled with negative control Donkey anti-mouse secondary antibody in CP. No AQ [file Image_2.tif]

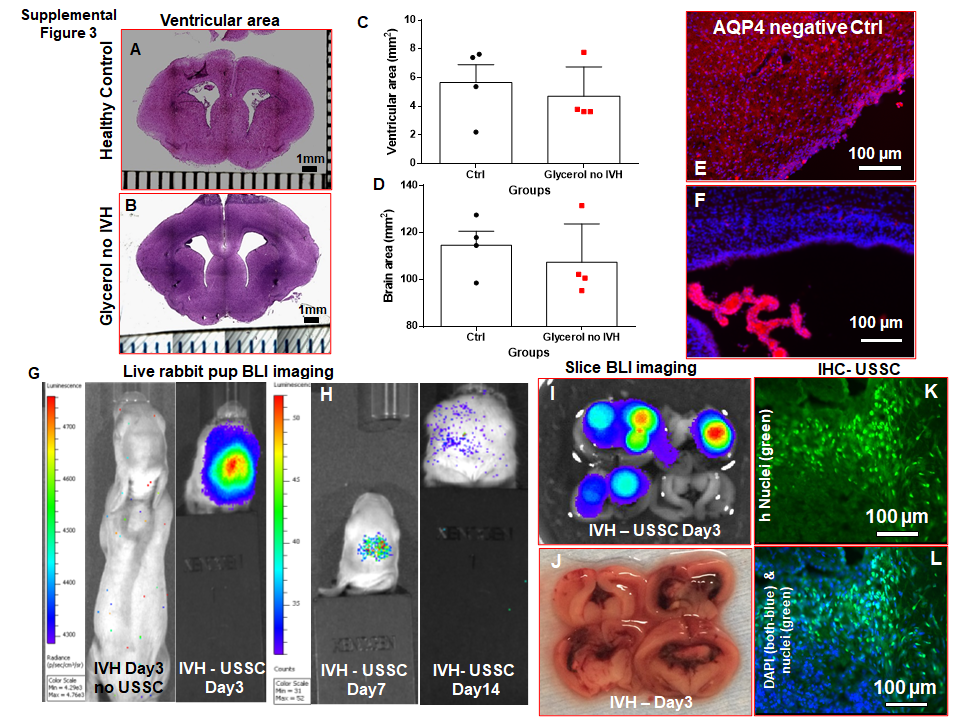

Supplement: SUPPLEMENTARY FIGURE 3 — (A,B) Representative hematoxylin and eosin (H&E) stained coronal section on postnatal day 14 at forebrain level-2 with and without glycerol injected no IVH pups. The total brain area and ventricular area was comparable in normal healthy control vs. glycerol injected no IVH control. The stitched images taken at low power for both postnatal ages; 20 μm sections. The scale bar is 1 mm. (C,D). Representative scatter plot with bar graphs shows cross-sectional area of lateral ventricles measurement (C) and total brain area (D) on postnatal day 14. The mean cross sectional measured on two alternate sections taken from the hippocampus towards the rostral side from the coronal block made at the level of the mid-septal nucleus (total ventricular area is sum of the left and right at level-2 and averaged for two alternate section for each pup). Each symbol in the experimental groups represent a rabbit pup (no significant differences in healthy control vs. glycerol no IIVH control; the values represent mean ± SEM; n = 4 in each group). (E,F) Representative immunofluorescence image of cryosections labeled with negative control for AQP4 Donkey anti-mouse secondary antibody in lateral ventricle wall. No AQP4 signal with secondary antibody alone cryosections stained on the lateral ventricle on the lateral side (E) and ventricle wall around the GM (F). Secondary antibody, Alexa-Flour 594, donkey anti-mouse (Cat #A21203, Invitrogen Thermo Fisher Scientific, Waltham, MA, USA). Highly cross-adsorbed secondary antibody with minimal cross reactivity to rabbit tissue. 20 μm sections. The scale bar is 100 μm. (G–J) Bioluminescence live image (BLI) of USSC administration by intracerebroventricular injection in IVH premature rabbit pups. (G) Representative BLI live image for IVH pup after single dose of USSC (right image) and no USSC injected negative control pup (left image). The strong red fluorescence signal indicates highest USSC density at postnatal day 3 (2 × 10^6^ cells/dose). (H) BLI im [file Image_3.tif]
